# The association between serum levels of oxLDL-lgG and oxLDL-lgM autoantibody with adult acute myeloblastic leukaemia

**DOI:** 10.1186/1476-511X-9-11

**Published:** 2010-01-31

**Authors:** Hao Li, Yu Tao Diao, Hui Qing Li, Qing Ma, Jia Cui, Ying Zhi Zhou, Dong Li

**Affiliations:** 1 Tumor Center of Qilu Hospital, Shandong University, Jinan, PR China; 2 College of Public Health, Shandong University, Jinan, PR China; 3 Institutes of Basic Medicine, Shandong Academy of Medical Sciences Jinan, PR China; 4 College of economics, Shandong University, Jinan, PR China; 5 Feicheng County People's Hospital, Feicheng, PR China

## Abstract

**Aim:**

To evaluate the relationship between serum antibodies against ox-LDL levels and adult acute myeloblastic leukemia (AML).

**Methods:**

Forty three patients with AML and 52 normal controls were enrolled in this study in the Department of Hematology, Tumor Center of Qilu Hospital of Shandong University from Feb. 2008 to Mar.2009. Serum lgG and lgM antibodies versus the oxLDL levels were evaluated by ELISA method. Data was analyzed by covariance and binary Logistic regression.

**Results:**

Serum mean levels of oxLDL-lgG in patients (38.92 ± 21.1259 ug/ml) were significantly lower than in control subjects (78.88 ± 9.3705 ug/ml); Meanwhile, Serum mean levels of oxLDL-lgM in patients (20.53 ± 10.2990 IU/L) were significantly higher than in control subjects (10.29 ± 10.5771 IU/L). Binary logistic regression showed the odds ratios of association of oxLD-lgG and oxLD-lgM with adult AML were 0.72(95%CI: 0.55-0.94) and 1.11(95%CI: 1.01-1.21) respectively after adjusted for potential confounders.

**Conclusion:**

In the preliminary investigation we found a descensive oxLDL- lgG and an elevated oxLDL-lgM serum levels for the adult AML. Future studies need to confirm the hypothesis whether they related to the development and progression of adult AML.

## Background

More than two decades ago, epidemiological studies showed a U-shaped relationship between total cholesterol (TC) levels and risk of all-cause mortality. The relationship between the baseline serum cholesterol level to total mortality was attributed to the high number of deaths associated with serum cholesterol level at the high end of the distribution (mainly due to coronary heart disease) and at the low end (mainly due to cancer) [1-3].

Recent studies showed that, as an endocrine organ, adipose tissue plays an important role in regulating energy metabolism and inflammation. It has also been associated with several cancers. Some studies have shown that adiponectin (ADP), Serum immunoglobulin G and immunoglobulin M antibodies versus the oxLDL levels (oxLDL-lgG, oxLDL-lgM) may play a role in the development and progression of various types of malignancies [4-6].

Moreover, there is no single published study with information on the serum levels of oxLDL-lgG and oxLDL-lgM for the patients with adult acute myeloblastic leukemia (AML). We made a primary study to evaluate the association of oxLD-lgG or oxLD-lgM with adult AML.

## Methods

### Study subjects

The present study covers 43 adult (17-70 years old) AML cases whose first diagnosis was performed based on clinical, laboratory, and blood smears of their bone marrow punctured in the Department of Hematology, Tumor Center of Qilu Hospital between February 2008 and March 2009.

Fifty-two controls were selected from a community screening examination of health care during the same period in the city. Patients with liver diseases, diabetes, or cardiovascular diseases including coronary heart disease, angina pectoris, myocardial infarction, cardiac arrhythmia, heart failure diagnosed via general medical check, electrocardiogram, and abdomen supersonic inspection were excluded from the study. Informed consent was obtained from all the subjects. The study protocol was approved by the local ethics committee.

### Measurements

A survey of the characteristics of the subjects such as name, gender, age, occupation, and so on was conducted using a questionnaire. Blood pressure was measured by a mercury sphygmomanometer. Hypertension was defined as medication dependent or systolic blood pressure (SBP) ≥140 mmHg and/or diastolic blood pressure (DBP) ≥90 mmHg.

### Blood sampling and biochemical analysis

Venous blood samples were taken from the study participants the morning after an overnight fast of at least 12 h. Plasma and serum were separated. The specimens were then kept frozen at -40°C until assayed. High-density lipoprotein-cholesterol (HDL-c), TC, and triglyceride (TG) levels were determined by enzymatic techniques. LDL-c was calculated by the Friedewald formula (LDL-c = TC - HDL-c - TG/5). Serum albumin was determined by the bromocresol green method.

### Determination of serum oxLDL, oxLDL-lgG, and oxLDL-lgM

An enzyme-linked immunosorbant assay (ELISA) for oxLDL, oxLDL-lgG, and oxLDL-lgM were performed with oxLDL, oxLDL-lgG, and oxLDL-lgM ELISA kits purchased from the Adliteram Diagnostic Laboratories, Inc., USA. According to the manufacturer's instructions, using coated microtitration strips of 96-well plates, plasma was diluted 1:1, and incubated at room temperature for 1 h in plates precoated with oxLDL, oxLDL-lgG, or oxLDL-lgM, respectively. After three washings, the plates were incubated with horseradish peroxidase (HRP) at room temperature for 30 minutes. After the removal of unbound conjugates by washing the samples three times, tetramethylbenzidine (TMB) was added to the wells as a chromogenic substrate. The mixture was incubated at room temperature in the dark for 10 minutes. Color development was stopped via a stopping solution, and absorbency was measured at 450 nm within 30 minutes. The oxLDL, oxLDL-lgG, and oxLDL-lgM titers were calculated by constructing a standard curve using the standards included in the kits. The oxLDL, oxLDL-lgG, and oxLDL-lgM concentrations in the samples were quantified in biomedical units as defined by the manufacturer. The intra-assay and inter-assay reproducibility (coefficients of variation) of the assay were 5%, 7%, and 10%, respectively.

### Statistical analysis

All statistical analyses were performed using SPSS15.0. The baseline characteristics were presented for quantitative data as mean ± standard deviation (SD), which compares the two groups with the Student's t test. Qualitative data were tested using the chi-square test. Correlations between variables were tested by Kendall's correlation test depending on the data distribution. Analysis of covariance (ANCOVA) was used to compare the oxLDL-lgG and oxLDL-lgM concentrations among two groups that control the influence of covariates. Binary logistic regression was applied to analyze the influencing factors. Probability was significant at a level of ≤0.05.

## Results

### Characteristics of subjects

Table 1 shows the clinical and biochemical characteristics of the studied population. No significant differences were observed for gender distribution, SBP, TG, and OXLDL. However, age, DBP, albumin, ADP, TC, HDL-c, LDL-c, oxLDL-lgG, and oxLDL-lgM were significantly different between the two groups.

**Table 1:** Characteristics of the Subjects

	Normal control (n = 52)	Adult AML (n = 43)	t-test	p
Sex, male(%)^a^	38.5	48.4	1.033	0.309
age	52.02 ± 12.269	42.72 ± 17.425	2.947	0.004
SBP mmHg	119.85 ± 12.432	125.12 ± 16.300	-1.742	0.085
DBP mmHg	78.17 ± 7.745	69.14 ± 12.1920	4.207	0.000
Albumin	45.75 ± 4.762	42.83 ± 6.729	2.455	0.017
TC mmol/L	5.50 ± 0.8123	4.01 ± 1.0569	7.796	0.000
TG mmol/L	1.42 ± 0.9356	1.65 ± 1.0770	-1.115	0.268
HDL-C mmol/L	1.22 ± 0.9280	0.62 ± 0.3982	4.192	0.000
LDL-C mmol/L	3.97 ± 1.1740	3.00 ± 1.0461	4.200	0.000
ADP ug/ml	4.21 ± 5.5410	7.56 ± 9.7684	-1.995	0.050
oxLDL ng/ml	34.74 ± 18.6549	31.34 ± 21.4124	0.882	0.413
oxLDL-IgG g/ml	78.88 ± 9.3705	38.92 ± 21.1259	11.486	0.000
oxLDL-IGM u/L	10.29 ± 10.5771	20.53 ± 10.2990	-4.756	0.000

Abbreviations: SBP, systolic blood pressure; DBP, diastolic blood pressure; TC, total cholesterol; TG, triglyceride; HDL-c, High density lipoprotein-cholesterol; LDL-c, Low density lipoprotein-cholesterol; ADP, adiponectin; oxLDL, oxidized low-density lipoprotein; oxLDL-lgG, immunoglobulin G type of autoantibodies to oxidized low-density lipoprotein; oxLDL-lgM, immunoglobulin M type of autoantibodies to oxidized low-density lipoprotein; AML, acute myeloblastic leukemia.

^a^: Pearson Chi-Square test.

### Comparison of oxLDL-lgG and oxLDL-lgM between two groups

Univariate analyses showed that plasma oxLDL-lgG correlated positively with age, DBP, TC, HDL-c, LDL-c, and oxLDL-lgM, but correlated negatively with TG. It showed no correlation to gender, SBP, albumin, HDL-c, and oxLDL. (Table 2). Meanwhile, plasma oxLDL-lgM correlated positively with ADP and negatively with age, DBP, TC, HDL-c, LDL-c, and oxLDL-lgG. It showed no correlation with sex, SBP, albumin, TG, and oxLDL.

**Table 2:** Influencing factors for oxLDL-lgG and oxLDL-lgM

Variables	oxLDL-lgG	oxLDL-lgM
sex	0.028	-0.027
age	0.172(**)	-0.102(*)
SBP	0.157(**)	-0.047
DBP	0.274(**)	-0.114(*)
Albumin	0.151(*)	-0,093
TC	0.308(**)	-0.225(**)
TG	-0.080	0.066
HDL-c	0.070	-0.105(*)
LDL-c	0.275(**)	-0.196(**)
ADP	-0.078	0.340(**)
OXLDL	0.104(*)	-0.096
oxLDL-lgG	1.000	-0.244(**)
oxLDL-lgM	-0.244(**)	1.000

Abbreviations: SBP, systolic blood pressure; DBP, diastolic blood pressure; TC, total cholesterol; TG, triglyceride; HDL-c, High density lipoprotein-cholesterol; LDL-c, Low density lipoprotein-cholesterol; ADP, adiponectin; oxLDL, oxidized low-density lipoprotein; oxLDL-lgG, immunoglobulin G type of autoantibodies to oxidized low-density lipoprotein; oxLDL-lgM, immunoglobulin M type of autoantibodies to oxidized low-density lipoprotein; AML, acute myeloblastic leukemia.

** Kendall's correlation is significant at the 0.01 level (2-tailed).* Correlation is significant at the 0.05 level (2-tailed).

As shown in Table 3 the oxLDL-lgG and oxLDL-lgM levels were significantly different between the two groups in ANCOVA models, which included the covariance variables of age, sex, SBP, DBP, albumin, LDL-c, HDL-c, oxLDL, ADP, TC, TG, and oxLDL-lgM in the model respectively.

**Table 3:** Estimated Marginal Means of oxLDL-lgG and oxLDL-lgM in Two Groups

	AML	controls		
			
	Mean ± SE	Mean ± SE	*F*	*P*
oxLDL-lgG ^a^	42.991 ± 3.833	74.787 ± 3.245	11.795	0.000
oxLDL-lgM ^b^	18.724 ± 2.050	12.003 ± 1.722	9.858	0.000

^a^: Covariates appearing in the covariance model are evaluated at the following variables: gender, age, SBP, DBP, albumin, TC, TG, HDL-c, LDL-c, ADP, oxLDL, and oxLDL-lgM.

^b^: Covariates appearing in the model are evaluated at the following variables: gender, age, SBP, DBP, albumin, TC, TG, HDL-c, LDL-c, ADP, oxLDL, and oxLDL-lgG.

### Association of oxLDL-lgG and oxLDL-lgM with AML

Table 4 shows that the odds ratios of association of oxLDL-lgG and oxLDL-lgM with adult AML are 0.72 (95% CI = 0.55, 0.94) and 1.11(95% CI = 1.01, 1.21) after adjusting for age, SBP, DBP, TC, HDL-c, and LDL-c, respectively.

**Table 4:** The association of oxLDL-lgG and oxLDL-lgM with adult AML

	OR	95%CI^a^	OR	95%CI^b^
oxLDL-IgG^c^	0.86	0.81-0.92	0.72	0.55-0.94
oxLDL-IGM^d^	1.09	1.05-1.14	1.11	1.01-1.21

Abbreviations: SBP, systolic blood pressure;DBP, diastolic blood pressure;TC, total cholesterol; HDL-c, High density lipoprotein-cholesterol; LDL-c, Low density lipoprotein-cholesterol; ADP, adiponectin; oxLDL, oxidized low-density lipoprotein; oxLDL-lgG, immunoglobulin G type of autoantibodies to oxidized low-density lipoprotein; oxLDL-lgM, immunoglobulin M type of autoantibodies to oxidized low-density lipoprotein; AML, acute myeloblastic leukemia; CI, Confidence Interval.

^a^:unadjusted odds ratio;

^b^: adjusted odds ratio

^c^: adjusted by age, SBP, DBP, albumin, oxLDL, LDL-c;

^d^: adjusted by age, DBP, TC, ADP, HDL-c and LDL-c.

## Discussion

The predominant isotype of oxLDL antibodies isolated either from serum (free antibodies) or precipitated soluble immune complexes (antigen-associated antibodies) is IgG of subclasses 1 and 3 [7]. At present, oxLDL antibodies have not yet become biomarkers for the development and/or progression of atherosclerosis [8]. Positive correlation between the levels of oxLDL-lgG antibodies and different endpoints considered as evidence of atherosclerotic vascular disease has been reported by many studies [9]. The protective role of IgM oxLDL antibodies has been proposed in human cardiovascular diseases [3].

In the present study, there were significantly lower serum levels of TC, HDL-c, and ADP in the AML group than that in the control group. The results were similar to those of a previous research [1-3,5].

The main finding of the present study was the descensive oxLDL-lgG and elevated oxLDL-lgM serum levels in patients with AML compared with normal controls after controlled potential confounders by the covariance analysis model. Logistic regression analysis revealed that lower oxLDL-lgG serum levels predict decreased risk of AML. However, higher oxLDL-lgM serum levels predict increased risk of AML. In contrast, there were both elevated oxLDL-lgG and descensive oxLDL-lgM serum levels in patients with cardiovascular or cerebrovascular diseases in the reported literature [3].

In recent years, there has been a growing body of evidence that excessive lipid peroxidation, including oxLDL and anti-oxLDL autoantibodies, which reflect in the indicators of oxidative stress *in vivo*, may play a key role in cancer development [9,10].

In this study, only selected adult AML patients were used as subjects. Lymphocytic leukemia and other blood diseases were ruled out, and the misclassification error does not exist. In the experimental determination of indicators, less than 10% coefficient of variation was the quality control. There were correlations between TC, TG, HDL, and LDL, resulting in a collinearity in the linear regression analysis. Consequently, a covariance of analysis was used in the study. Therefore, the results of this study are not biased.

## Conclusion

The present study shows that in contrast with the detecting antibodies of oxLDL-lgG and oxLDL-lgM serum levels in patients with cardiovascular and cerebrovascular diseases in literature, the descensive oxLDL-lgG and the elevated oxLDL-lgM serum levels may be related to the development and progression of adult AML. However, the findings are the results of a preliminary investigation. Many future studies are needed to answer: what is a biological mechanism/relationship behind the findings? Is IgG anti-oxLDL lower in patients because it is complexes/bound to oxLDL? Is IgG anti-oxLDL thought as a cause linkage with the development of AML biology and leukemogenesis? How about the relationship to other known risk factors for AML such as smoking, exposure to certain chemicals etc? Is lipid peroxidation and oxidative stress pronounced in AML? Is there a relationship between anti-oxLDL and the stage of the disease? After that the hypothesis of whether oxLDL-lgG as well as oxLDL-lgM plays a causative or merely consequential role in AML can be verified, and in the interim, a novel strategy of prevention and therapy for AML can be designated.

## Abbreviations

TC: total cholesterol; TG: triglyceride; HDL-c: High density lipoprotein-cholesterol; LDL-c: Low density lipoprotein-cholesterol; oxLDL: oxidized low-density lipoprotein; oxLDL-lgG: immunoglobulin G type of autoantibodies to oxidized low-density lipoprotein; oxLDL-lgM: immunoglobulin M type of autoantibodies to oxidized low-density lipoprotein; ADP: adiponectin; AML:acute myeloblastic leukamia; SBP: systolic blood pressure; DBP: diastolic blood pressure; ELISA: enzyme-linked immunosorbant assay.

## Competing interests

The authors declare that they have no competing interests.

## Authors' contributions

Members listed below made their respective contributions to this manuscript. Professor HL designed the skeleton of this study, supervised the epidemiologic survey of the characteristics of the subjects, performed the statistical analysis and drafted the manuscript. YTD, HQL, QM, JC, YZZ and DL carried out the immunoassays in addition to biochemical analysis. All authors read and approved the final manuscript.
